# A Common Variant in the SETD7 Gene Predicts Serum Lycopene Concentrations

**DOI:** 10.3390/nu8020082

**Published:** 2016-02-06

**Authors:** Christopher R. D’Adamo, Antonietta D’Urso, Kathleen A. Ryan, Laura M. Yerges-Armstrong, Richard D. Semba, Nanette I. Steinle, Braxton D. Mitchell, Alan R. Shuldiner, Patrick F. McArdle

**Affiliations:** 1 Department of Family & Community Medicine, University of Maryland School of Medicine, Baltimore, MD 21201, USA; 2 The Commonwealth Medical College, Scranton, PA 18509, USA; adurso@tcmc.edu; 3 Department of Medicine, University of Maryland School of Medicine, Baltimore, MD 21201, USA; kryan@medicine.umaryland.edu (K.A.R.); lyerges@medicine.umaryland.edu (L.M.Y.-A.); nsteinle@medicine.umaryland.edu (N.I.S.); bmitchel@medicine.umaryland.edu (B.D.M.); ashuldin@medicine.umaryland.edu (A.R.S.); pmcardle@medicine.umaryland.edu (P.F.M.); 4 Wilmer Eye Institute, Johns Hopkins University School of Medicine, Baltimore, MD 21287, USA; rdsemba@jhmi.edu

**Keywords:** lycopene, carotenoids, genome-wide association study (GWAS), SETD7, SCARB1, Old Order Amish, prostate cancer

## Abstract

Dietary intake and higher serum concentrations of lycopene have been associated with lower incidence of prostate cancer and other chronic diseases. Identifying determinants of serum lycopene concentrations may thus have important public health implications. Prior studies have suggested that serum lycopene concentrations are under partial genetic control. The goal of this research was to identify genetic predictors of serum lycopene concentrations using the genome-wide association study (GWAS) approach among a sample of 441 Old Order Amish adults that consumed a controlled diet. Linear regression models were utilized to evaluate associations between genetic variants and serum concentrations of lycopene. Variant rs7680948 on chromosome 4, located in the intron region of the *SETD7* gene, was significantly associated with serum lycopene concentrations (*p* = 3.41 × 10^−9^). Our findings also provided nominal support for the association previously noted between *SCARB1* and serum lycopene concentrations, although with a different SNP (rs11057841) in the region. This study identified a novel locus associated with serum lycopene concentrations and our results raise a number of intriguing possibilities regarding the nature of the relationship between *SETD7* and lycopene, both of which have been independently associated with prostate cancer. Further investigation into this relationship might help provide greater mechanistic understanding of these associations.

## 1. Introduction

The carotenoids are a group of over 600 pigments that are synthesized by plants and microorganisms. Lycopene is a red-pigmented carotenoid present in tomatoes, watermelon, papaya, and other fruits and vegetables. There is no endogenous production of lycopene or other carotenoids in animals and they are only obtained from the diet. Higher intake and circulating concentrations of carotenoids have been associated with lower risk of cancer [1], cardiovascular disease [2,3], metabolic syndrome [4], and diseases of the eye [5]. The protective effects of dietary carotenoids appear to be due in part to their antioxidant activity. Lycopene has among the most potent antioxidant effects of the dietary carotenoids and is distinct from some other carotenoids because it does not form vitamin A [6]. Interventions utilizing lycopene-rich diets and lycopene supplementation have revealed reductions in inflammation, oxidative stress, markers of atherosclerosis, and increases in high density lipoprotein (HDL) [7,8]. Meta-analysis of intervention trials has also revealed that lycopene supplementation reduces serum cholesterol and blood pressure [9].

Prostate cancer is the disease that has been studied most extensively in relation to lycopene. While the evidence is not conclusive, a number of large epidemiologic studies indicate that higher dietary intake and serum concentrations of lycopene are associated with lower risk of prostate cancer [10,11,12], particularly lethal prostate cancer [13]. Lycopene supplementation has also been shown to inhibit the progression of benign prostate hyperplasia [14]. It has also been proposed that serum concentrations of lycopene and other carotenoids may be useful in the early detection of prostate cancer [15,16]. There are three chief proposed target pathways for lycopene’s involvement in prostate cancer: antioxidant and anti-inflammatory function, hormonal modulation, and epigenetic modification [17,18,19,20]. Lycopene appears as if it may play a role in prostate cancer, but there have also been a number of large studies that have found no relationship between serum concentrations of lycopene and risk of prostate cancer [21,22]. Thus, the relationship between both dietary intake and circulating concentrations of lycopene and prostate cancer is not fully understood and there has been a call for further study in this area [11].

Further confounding the relationship between lycopene and human disease is the relatively poor correlation between dietary intake of lycopene in isolation and its concentrations in serum [23]. The absorption, bioavailability, and serum concentrations of lycopene vary substantially due to other accompanying dietary factors. For instance, consuming fat along with lycopene greatly increases circulating concentrations of this lipid-soluble nutrient [24]. Genetic variants may also play a role in how carotenoids are absorbed, transported, or metabolized and could help explain the discord between dietary intake and serum concentrations of lycopene. Previous candidate gene studies have identified genetic variants associated with circulating lycopene concentrations. Assessment of single nucleotide polymorphisms (SNPs) using candidate genes yielded variants in intestinal fatty acid binding protein, a cytoplasmic protein that transports long chain fatty acids [25], as well as Apo A-IV and Apo B, proteins involved in lipoprotein metabolism [26].

There are two previously reported genome-wide association studies (GWAS) of lycopene. The first, a population based study in the Tuscany Chianti region of Italy, did not identify any genome-wide significant associations with lycopene [27]). In the other, three genetic loci were identified as associated with circulating lycopene concentrations in a multi-ethnic population of women [28]. SNPs in the gene *SCARB1*, which encodes for a cholesterol membrane transporter, were genome-wide significant in the multi-ethnic meta-analysis, though the signal was driven by participants of Hispanic and African heritage primarily. The other two associated loci in *SLT3* and *DHRS2*, were identified in African-Americans only. No genome-wide significant associations have been identified in populations of European descent.

The main aim of this study was to identify novel genetic predictors of serum lycopene concentrations. We studied a population of Old Order Amish adults living in Lancaster County, Pennsylvania in whom serum lycopene concentrations were measured at the conclusion of a controlled diet. The Amish are a commonly studied population for these types of studies because, among other reasons, their relatively homogeneous genetics and lifestyles across the population increase the ability to detect genetic signals [29,30,31,32].

## 2. Experimental Section

### 2.1. Study Population

The study sample was composed of 441 Caucasian participants from the Heredity and Phenotype Intervention (HAPI) Heart Study who completed a 6-day controlled diet and from whom frozen blood samples were available for serum lycopene measurement. The HAPI Heart Study is a family study and by design included individuals from the same nuclear family. Moreover, many of the enrolled families were related to each other given the social structure of the Old Order Amish community. The design of the HAPI Heart Study, including study inclusion/exclusion criteria, demographics of the full study sample, *etc.*, has been described previously [33] and was registered on Clinicaltrials.gov (NCT00664040). Of the 868 Old Order Amish adults recruited into the HAPI Heart study, 469 were administered the controlled dietary intervention and subsequently provided a fasting blood sample at the conclusion of the 6-day diet. There were 27 samples of insufficient quality to measure serum lycopene concentrations and one sample excluded from analysis due to extreme serum lycopene concentration (22,995 μg/dL) that was considered an outlier. There were 308 nuclear families in our study sample, ranging from one participant to nine participants per family. The participant relatedness was as follows: 113 parent-children, 198 sibling, 105 avuncular, 33 first cousins, and six grandparents-grandchildren. The study was approved by the Institutional Review Board of the University of Maryland School of Medicine and all participants provided written informed consent.

### 2.2. Controlled Diet

A controlled diet was prepared for participants by study staff. A registered dietitian visited several Old Order Amish households to obtain diet histories and observe meals and foods that were in their homes. All meals in the controlled diet were designed to be representative of the typical diets of Old Order Amish adults and were provided to the study participants by home delivery over a period of six days. Study participants also abstained from both prescribed and over the counter medications and dietary supplements during this 6-day period. The full menus for the 6-day controlled diet that the participants in this study consumed are provided in the Supplementary Materials. The controlled diet contained an average of 3277 kilocalories per day; 49% from carbohydrate, 15% from protein, and 36% from fat. There was an average of 525 mg of cholesterol per day in the diet. While designed to be representative of the typical diet of the Old Order Amish, the controlled diet was higher in carbohydrate, total fat, and cholesterol than has generally been suggested in the Dietary Guidelines for Americans of the United States Department of Agriculture. The diet contained approximately 10.4 mg of lycopene per day, coming primarily from tomatoes and tomato sauce.

Compliance with the controlled diet was assessed by comparing sodium, potassium, and creatinine levels from first morning urine samples obtained: (1) prior to consuming the 6-day controlled diet that participants consumed in this study; (2) on the final day of the 6-day controlled diet that the participants consumed in this study; and (3) on the final day of a second isocaloric, 6-day controlled diet that was low in salt and consumed after the blood draw that was used to conduct the GWAS in this study. The compliance data have been reported in detail previously [34], but in brief, the excreted sodium, potassium, and creatinine levels that reflect varying salt content of the diets revealed high compliance with the controlled diet in this study.

### 2.3. Serum Lycopene Measurement

Frozen blood samples that had been obtained after a 12-h fast were assayed for serum lycopene concentrations at Johns Hopkins University. 200 μL from each frozen blood sample were used for the reverse-phase high-pressure liquid chromatography (HPLC) assessment of serum lycopene concentrations [35]. There were 13 batches run and the intra-assay and inter-assay coefficients of variation CVs for lycopene were 7.9% and 17.4%, respectively.

### 2.4. Genotyping

A genome-wide association study assesses associations of phenotype with single nucleotide polymorphisms (SNPs) throughout the genome. Study participants were genotyped using either the Affymetrix 500k or Affymetrix 1M SNP chip v6.0 by the Genomics Core Laboratory at the University of Maryland. Genotyping calls were made using Birdseed, which is part of the Birdsuite tools [36]. A total of 397,704 SNPs were in common on both arrays and used for analysis. SNPs with a minor allele frequency (MAF) ≥ 1%, a call rate exceeding 95% and conforming to the expectations of Hardy-Weinberg equilibrium (*p* > 10^−6^) were used for imputation with MACH using the HapMap CEU reference sample [37]. Results were filtered using a MAF ≥ 2% and an imputation quality score ≥ 30%, for a final analyzed SNP count of 2,302,013.

### 2.5. Statistical Methods

Descriptive statistics were performed to characterize the study sample and determine the mean concentrations of serum lycopene. For GWAS analysis, we estimated the effect of genotype on lycopene levels, adjusting for the effects of age, sex, and body-mass index (BMI) using a general linear model. Genotype was coded as the number of copies of the reference allele (0, 1, or 2), thus corresponding to an additive genetic model. The GWAS analyses were performed using the MMAP software [38], which accounts for family structure as a random effect. Statistical analysis was performed using a variance component approach to account for relatedness among study participants. This approach has previously been shown to provide valid estimates of regression parameters [39]. To account for the multiple SNPs tested, we considered associations at *p* < 5 × 10^−8^ to be statistically significant. At this genome-wide significance threshold, we estimated that our sample provided 80% power to detect SNPs accounting for 9%–10% of trait variation.

## 3. Results

Baseline characteristics of the study sample are provided in Table 1. There were more men in the study than women (254 men, 187 women). Participants were in their mid-40s on average (mean = 43.1 years) with the men being slightly younger than the women. Participants had a mean BMI of 26.4 kg/m^2^ and over half of both the men and the women could be classified as overweight (BMI ≥ 25 kg/m^2^). Mean lycopene values were 39.2 μg/dL (standard deviation = 19.9 μg/dL, standard error of mean = 10.7 μg/dL, range = (7.5–136.9 μg/dL)). The heritability was estimated to be 0.38 ± 0.12.

**Table 1:** Characteristics of Old Order Amish study sample from Lancaster County, Pennsylvania after consuming a 6-day controlled diet.

Characteristic	All (*n* = 441)	Female (*n* = 187)	Male (*n* = 254)
Age (years)	43.1 (13.0)	45.7 (13.2)	41.2 (12.5)
BMI (kg/m^2^)	26.4 (4.24)	27.8 (5.09)	25.4 (3.12)
Lycopene (μg/dL)	39.2 (19.9)	37.6 (17.7)	39.7 (21.5)
Retinol (μg/dL)	44.1 (10.6)	43.5 (10.9)	44.4 (10.3)
Lutein (μg/dL)	14.2 (5.7)	13.1 (5.1)	15.4 (5.7)
Zeaxanthin (μg/dL)	6.8 (3.4)	6.3 (2.8)	7.4 (3.4)
β-Cryptoxanthin (μg/dL)	8.8 (3.9)	8.8 (3.9)	8.8 (3.3)
α-Carotene (μg/dL)	15.6 (12.3)	17.2 (13.4)	14.5 (11.3)
β-Carotene (μg/dL)	37.6 (26.8)	41.9 (31.1)	34.9 (23.1)
γ-Tocopherol (μg/dL)	194.8 (69.1)	195.7 (74.1)	194.0 (65.8)
α-Tocopherol (μg/dL) 0.02322	1309.2 (335.1)	343.7 (346.3)	283.4 (325.2)

**Figure 1:**
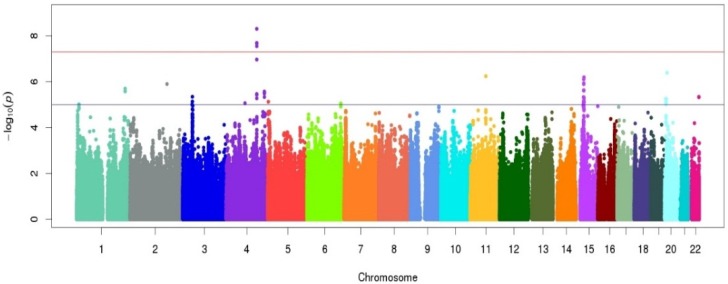
Manhattan plot for the genome-wide association study (GWAS) of serum lycopene concentrations in the Old Order Amish study population following a 6-day controlled diet. The x-axis represents chromosomal position along the genome. The y-axis shows the *p*-value for association test at each locus on the log scale. The colors are used to identify the different chromosomes listed on the x-axis.

**Figure 2:**
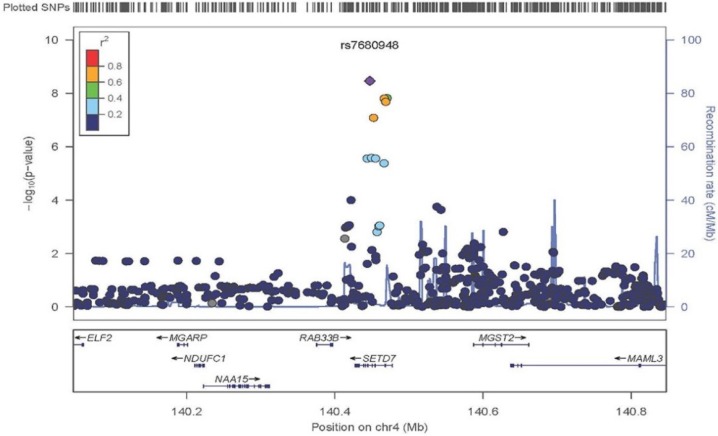
Regional association plot of chromosome 4q31.1 in the area of *SETD7*. Variant rs7680948 provides genome-wide significant evidence of association with serum lycopene concentrations. The x-axis represents chromosomal position on chromosome 4 with the location of genes at the locus annotated. The left y-axis shows the *p*-value for association tests at each locus (dot) on the log scale. The right y-axis provides recombination rates in centimorgans per megabase in the chromosomal region identifying recombination hotspots in the region (grey line). The diamond is the “top hit” (*i.e.*, the strongest association). Other SNPs in the region are represented by circles. The colors indicate linkage disequilibrium per the r^2^ map on top left. Linkage disequilibrium associated with the top signal appears to span the entire region of gene *SETD7*.

**Figure 3:**
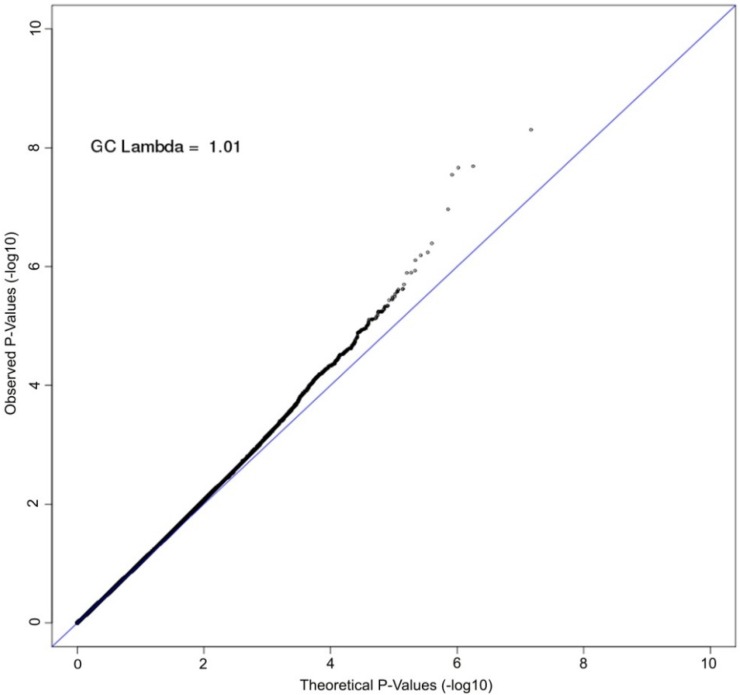
Quantile-quantile (QQ) plot of GWAS of serum lycopene concentrations. The axes plot the observed (y-axis) *vs.* theoretical (x-axis) association *p*-values on the log scale for all single nucleotide polymorphisms (SNPs) with minor allele frequency (MAF) greater than 2%. The Old Order Amish are a closed founder populations with little admixture expected. The genomic control lambda is estimated to be 1.01, indicating little bias due to population stratification.

A Manhattan plot summarizing results of the GWAS is provided in Figure 1. The top hits from the association analyses are presented in Table 2. We detected genome-wide significant evidence for association of lycopene levels to a locus on chromosome 4q31 (lead SNP = rs7680948; age, sex, and BMI-adjusted *p* = 3.41 × 10^−9^). Each copy of the A allele was associated with a 8.6 μg/dL decrease in serum lycopene, and this locus accounted for 9.3% of the variation in lycopene levels. The minor allele at the locus (A) is common in the Old Order Amish (MAF = 0.202) as well as other populations (Hapmap CEU = 0.265, Hapmap YRI = 0.394). Figure 2 provides a regional association plot of the genome-wide significant association on chromosome 4. Rs7680948 is in an intronic region of gene *SETD7*. Gene *SETD7* encodes the enzyme histone H3-K4 methyltransferase SETD7, one of the histone methyltransferases (HMTs) enzymes. The quantile-quantile plot provided in Figure 3 reveals little evidence for genomic inflation (lambda = 1.01), as the observed distribution of *p*-values for the genome-wide association tests is consistent with that expected under the null.

**Table 2:** Genome-wide (*p* < 5 × 10^−8^) and sub genome-wide (5 × 10^−8^ ≤ *p* < 1 × 10^−6^) associations with serum lycopene concentrations.

SNP	Chromosome	Position	Gene	MAF	Coded Allele	Beta (SE)	*p*-Value
*Genome-wide Significant*							
rs7680948	4	140447105	*SETD7*	0.20	A	−0.19 (0.03)	4.97 × 10^−9^
*Sub Genome-wide Significant*							
rs4635297	15	38327408	*BC039545*	0.08	A	0.26 (0.05)	6.46 × 10^−7^
rs341075	11	71935611	–	0.02	A	−0.87 (0.17)	5.75 × 10^−7^
rs6108801	20	10989519	*–*	0.04	C	−0.48 (0.09)	4.07 × 10^−7^
rs2232315	2	169757432	*G6PC2*	0.03	A	0.74 (0.15)	1.26 × 10^−6^

We additionally identified three other loci for which associations were observed at *p* < 1 × 10^−6^ that are provided in Table 2. We also performed look-ups for SNPs previously reported to be associated with serum lycopene levels in a multiethnic GWAS [28], in which associations were reported at three loci: One achieving genome-wide association to three SNPs in high linkage disequilibrium in *SCARB1* (lead SNP: rs1672879) in the meta-analysis across three ethnic groups, and the other two achieving genome-wide significance in the African-American sample only (to SNPs in *SLIT3* (lead SNP: rs11057841) and *DHRS2* (lead SNP: rs74036811)). Notably, the associated SNPs in *SCARB1* have a minor allele frequency (MAF) of only 0.03 in European-Americans and the associated SNPs in *SLIT3* and *DHRS2* were monomorphic in European-Americans. In the Amish, the MAF of the three SNPs were even lower (MAF = 0.015), and there was no evidence for association with lycopene levels (*p* = 0.26). Although not a replication, we did, however, detect nominal evidence for association of lycopene levels with a different SNP in *SCARB1* (rs11057841; MAF = 0.14; *p* = 3.79 × 10^−4^).

## 4. Discussion

The key result from this study is the novel association observed between a common variant at the *SETD7* locus, rs7680948, and serum lycopene concentrations in this genome-wide association study. This represents the first genome-wide significant genetic association of lycopene in a mixed-gender, Caucasian population and the first study evaluating genetic determinants of lycopene concentrations among a sample that had consumed a controlled diet.

We were unable to replicate an association previously noted between a SNP in *SCARB1* with low MAF in Caucasians and serum lycopene concentrations, although we did observe a nominal association of lycopene levels with a different SNP within this gene (rs11057841). Interestingly, rs11057841 has previously been associated with lipoprotein-associated phospholipase A_2_ (Lp-PLA_2_) [40]. Both Lp-PLA_2_ and lycopene are primarily carried throughout circulation on low-density lipoprotein (LDL) [41,42]. Our data do not provide replicative support for either *SLIT3* or *DHRS2* [28], although this is not surprising as these prior associations were detected to a SNP found only in African-Americans and not in Caucasian Americans of European descent.

Our results raise a number of intriguing possibilities regarding the nature of the relationships previously noted between *SETD7*, lycopene, and prostate cancer. *SETD7* is proliferative and anti-apoptotic in prostate cancer cells and nuclear expression is upregulated in prostate cancer tissue [43]. The activity of *SETD7* as a histone methyltransferase (HMT) may also play a role in prostate cancer. HMTs have been shown to be upregulated in prostate cancer [44,45] and deregulation of HMTs has also been associated with prostate cancer development and progression [46]. It is also plausible that *SETD7* may be related to prostate cancer risk through its relationship to serum lycopene concentrations, as was identified in this study. The potential protective mechanisms of lycopene against prostate cancer include regulation of the antioxidant response element, exertion of effects on VEGF signaling pathways, induction of cell cycle arrest, and mediation of apoptosis [18,47]. Future studies containing prostate cancer endpoints would be necessary to confirm this relationship.

There are several key strengths of the study. This was the first GWAS aimed at identifying genetic predictors of serum lycopene concentrations that was conducted among a sample that had consumed a controlled diet. The controlled diet and consistent lycopene, fat, and cholesterol intake among the study participants enabled us to more closely isolate the genetic contributions to the variance in serum lycopene than would have been possible on a variable diet. A related strength was that adherence to the diet was also high, as verified by urinary excretion. The diet was informed by home visits of the study population performed by a registered dietitian, was designed to be culturally-appropriate based upon the foods and beverages present in the homes during these visits, and was delivered to the homes of the study participants to encourage adherence. Conduct of this study in the Old Order Amish population was also advantageous for several reasons. To our knowledge, this is the first study to estimate heritability of serum lycopene concentrations in humans, an analysis made possible by the relationship structure of the Old Order Amish. The Old Order Amish study sample also provided a population that was relatively homogenous with respect to genetics, environmental exposures, and lifestyle habits. This homogeneity, particularly with respect to genetics, provided increased power to detect genetic variants associated with lycopene concentrations.

There were also several notable limitations to this study. The relatively small sample size (*n* = 441) may have limited our ability to detect genome-wide significant associations between genetic variants and serum lycopene concentrations. Furthermore, the relatively high inter-assay CV of 17.4% for our serum lycopene measurements could have resulted in lower precision of our estimates. However, despite the relatively small sample size and relatively high inter-assay CV, our study was able to identify a novel locus associated with serum lycopene concentrations. We attribute this success in part to the aforementioned advantages of studying an Old Order Amish population as well as the controlled diet that the participants consumed prior to the fasted-state blood draw which enabled us to more closely isolate the genetic contributions to serum lycopene concentrations. A limitation of the controlled diet was its relatively short duration of six days. While the time to maximum concentration of lycopene after consumption is just six hours, lycopene has an elimination half-life of between five and nine days [48,49]. It is likely that the serum lycopene concentrations measured at the conclusion of the controlled diet were also influenced to some degree by variable dietary intake that occurred prior to the initiation of the controlled diet. However, the controlled diet was designed to be representative of the typical Old Order Amish diet and to the authors’ knowledge, all previously published GWAS of lycopene and carotenoid concentrations have been conducted among populations on uncontrolled diets. Thus, we do not believe that this limitation of the controlled diet has a major influence on the findings of this study. Finally, while the novel association noted between a variant in *SETD7* and serum lycopene concentrations, both of which have been associated with prostate cancer, may provide the rationale for further study into the specific mechanisms of this relationship, this study did not collect data on family history of prostate cancer, prostate specific antigen, or other markers of the disease and no direct inference can be made.

In conclusion, this study provides the identification of a novel genetic association between rs7680948, an intronic variant in *SETD7*, and serum lycopene concentrations. These findings provide further support that genetics may affect serum concentrations of lycopene. Further studies are needed to clarify any potential relationships between *SETD7*, lycopene, and clinical endpoints such as prostate cancer.

## Supplementary Materials

The following are available online at http://www.mdpi.com/2072-6643/8/2/82/s1.

## References

[1] Nishino H., Murakosh M., Ii T., Takemura M., Kuchide M., Kanazawa M., Mou X.Y., Wada S., Masuda M., Ohsaka Y. (2002). Carotenoids in cancer chemoprevention. Cancer Metastasis Rev..

[2] Wang Y., Chung S.J., McCullough M.L., Song W.O., Fernandez M.L., Koo S.I., Chun O.K. (2014). Dietary carotenoids are associated with cardiovascular disease risk biomarkers mediated by serum carotenoid concentrations. J. Nutr..

[3] Ciccone M.M., Cortese F., Gesualdo M., Carbonara S., Zito A., Ricci G., De Pascalis F., Scicchitano P., Riccioni G. (2013). Dietary intake of carotenoids and their antioxidant and anti-inflammatory effects in cardiovascular care. Mediat. Inflamm..

[4] Beydoun M.A., Canas J.A., Beydoun H.A., Chen X., Shroff M.R., Zonderman A.B. (2012). Serum antioxidant concentrations and metabolic syndrome are associated among U.S. adolescents in recent national surveys. J. Nutr..

[5] Van Leeuwen R., Boekhoorn S., Vingerling J.R., Witteman J.C., Klaver C.C., Hofman A., de Jong P.T. (2005). Dietary intake of antioxidants and risk of age-related macular degeneration. JAMA.

[6] Rao A.V., Rao L.G. (2007). Carotenoids and Human Health. Pharmacol. Res..

[7] McEneny J., Wade L., Young I.S., Masson L., Duthie G., McGinty A., McMaster C., Thies F. (2013). Lycopene intervention reduces inflammation and improves HDL functionality in moderately overweight middle-aged individuals. J. Nutr. Biochem..

[8] Kim J.Y., Paik J.K., Kim O.Y., Park H.W., Lee J.H., Jang Y., Lee J.H. (2011). Effects of lycopene supplementation on oxidative stress and markers of endothelial function in healthy men. Atherosclerosis.

[9] Ried K., Fakler P. (2011). Protective effect of lycopene on serum cholesterol and blood pressure: Meta-analyses of intervention trials. Maturitas.

[10] Wei M.Y., Giovannucci E.L. (2012). Lycopene, Tomato products, and prostate cancer incidence: A review and reassessment in the PSA screening era. J. Oncol..

[11] Ilic D., Forbes K.M., Hassed C. (2011). Lycopene for the prevention of prostate cancer. Cochrane Database Syst. Rev..

[12] Giovannucci E. (2002). A review of epidemiologic studies of tomatoes, lycopene, and prostate cancer. Exp. Biol. Med..

[13] Zu K., Mucci L., Rosner B.A., Clinton S.K., Loda M., Stampfer M.J., Giovannucci E. (2014). Dietary lycopene, angiogenesis, and prostate cancer: A prospective study in the prostate-specific antigen era. J. Natl. Cancer Inst..

[14] Schwarz S., Obermuller-Jevic U.C., Hellmis E., Koch W., Jacobi G., Biesalski H.K. (2008). Lycopene inhibits disease progression in patients with benign prostate hyperplasia. J. Nutr..

[15] Beydoun H.A., Shroff M.R., Mohan R., Beydoun M.A. (2011). Associations of serum vitamin a and carotenoid levels with markers of prostate cancer detection among U.S. men. Cancer Causes Control.

[16] Beilby J., Ambrosini G.L., Rossi E., de Klerk N.H., Musk A.W. (2010). Serum levels of folate, lycopene, β-carotene, retinol and vitamin e and prostate cancer risk. Eur. J. Clin. Nutr..

[17] Chen J., Song Y., Zhang L. (2013). Effect of lycopene supplementation on oxidative stress: An exploratory systematic review and meta-analysis of randomized controlled trials. J. Med. Food.

[18] Trejo-Solis C., Pedraza-Chaverri J., Torres-Ramos M., Jimenez-Farfan D., Cruz Salgado A., Serrano-Garcia N., Osorio-Rico L., Sotelo J. (2013). Multiple molecular and cellular mechanisms of action of lycopene in cancer inhibition. Evid. Based Alternat Med..

[19] Wang X.D. (2012). Lycopene metabolism and its biological significance. Am. J. Clin. Nutr..

[20] Palozza P., Parrone N., Catalano A., Simone R. (2010). Tomato lycopene and inflammatory cascade: Basic interactions and clinical implications. Curr. Med. Chem..

[21] Peters U., Leitzmann M.F., Chatterjee N., Wang Y., Albanes D., Gelmann E.P., Friesen M.D., Riboli E., Hayes R.B. (2007). Serum lycopene, other carotenoids, and prostate cancer risk: A nested case-control study in the prostate, lung, colorectal, and ovarian cancer screening trial. Cancer Epidemiol. Biomark. Prev..

[22] Kristal A.R., Till C., Platz E.A., Song X., King I.B., Neuhouser M.L., Ambrosone C.B., Thompson I.M. (2011). Serum lycopene concentration and prostate cancer risk: Results from the prostate cancer prevention trial. Cancer Epidemiol. Biomark. Prev..

[23] Jenab M., Ferrari P., Mazuir M., Tjonneland A., Clavel-Chapelon F., Linseisen J., Trichopoulou A., Tumino R., Bueno-de-Mesquita H.B., Lund E. (2005). Variations in lycopene blood levels and tomato consumption across European countries based on the European prospective investigation into cancer and nutrition (EPIC) study. J. Nutr..

[24] Brown M.J., Ferruzzi M.G., Nguyen M.L., Cooper D.A., Eldridge A.L., Schwartz S.J., White W.S. (2004). Carotenoid bioavailability is higher from salads ingested with full-fat than with fat-reduced salad dressings as measured with electrochemical detection. Am. J. Clin. Nutr..

[25] Borel P., Moussa M., Reboul E., Lyan B., Defoort C., Vincent-Baudry S., Maillot M., Gastaldi M., Darmon M., Portugal H. (2009). Human fasting plasma concentrations of vitamin e and carotenoids, and their association with genetic variants in Apo C-III, cholesteryl ester transfer protein, hepatic lipase, intestinal fatty acid binding protein and microsomal triacylglycerol transfer protein. Br. J. Nutr..

[26] Borel P., Moussa M., Reboul E., Lyan B., Defoort C., Vincent-Baudry S., Maillot M., Gastaldi M., Darmon M., Portugal H. (2007). Human plasma levels of vitamin e and carotenoids are associated with genetic polymorphisms in genes involved in lipid metabolism. J. Nutr..

[27] Ferrucci L., Perry J.R., Matteini A., Perola M., Tanaka T., Silander K., Rice N., Melzer D., Murray A., Cluett C. (2009). Common variation in the β-carotene 15,15′-monooxygenase 1 gene affects circulating levels of carotenoids: A genome-wide association study. Am. J. Hum. Genet..

[28] Zubair N., Kooperberg C., Liu J., Di C., Peters U., Neuhouser M.L. (2015). Genetic variation predicts serum lycopene concentrations in a multiethnic population of postmenopausal women. J. Nutr..

[29] Shen H., Damcott C.M., Rampersaud E., Pollin T.I., Horenstein R.B., McArdle P.F., Peyser P.A., Bielak L.F., Post W.S., Chang Y.P. (2010). Familial defective apolipoprotein B-100 and increased low-density lipoprotein cholesterol and coronary artery calcification in the Old Order Amish. Arch. Intern. Med..

[30] Shuldiner A.R., O’Connell J.R., Bliden K.P., Gandhi A., Ryan K., Horenstein R.B., Damcott C.M., Pakyz R., Tantry U.S., Gibson Q. (2009). Association of cytochrome P450 2C19 genotype with the Antiplatelet effect and clinical efficacy of clopidogrel therapy. JAMA.

[31] McArdle P.F., Parsa A., Chang Y.P., Weir M.R., O’Connell J.R., Mitchell B.D., Shuldiner A.R. (2008). Association of a common nonsynonymous variant in GLUT9 with serum uric acid levels in Old Order Amish. Arthritis Rheum..

[32] Pollin T.I., Damcott C.M., Shen H., Ott S.H., Shelton J., Horenstein R.B., Post W., McLenithan J.C., Bielak L.F., Peyser P.A. (2008). A Null mutation in human APOC3 confers a favorable plasma lipid profile and apparent cardioprotection. Science.

[33] Mitchell B.D., McArdle P.F., Shen H., Rampersaud E., Pollin T.I., Bielak L.F., Jaquish C., Douglas J.A., Roy-Gagnon M.H., Sack P. (2008). The genetic response to short-term interventions affecting cardiovascular function: rationale and design of the heredity and phenotype intervention (HAPI) heart study. Am. Heart J..

[34] Montasser M.E., Douglas J.A., Roy-Gagnon M.H., Van Hout C.V., Weir M.R., Vogel R., Parsa A., Steinle N.I., Snitker S., Brereton N.H. (2011). Determinants of blood pressure response to low-salt intake in a healthy adult population. J. Clin. Hypertens..

[35] Sowell A.L., Huff D.L., Yeager P.R., Caudill S.P., Gunter E.W. (1994). Retinol, α-Tocopherol, lutein/zeaxanthin, β-Cryptoxanthin, Lycopene, α-carotene, Trans-β-carotene, and four retinyl esters in serum determined simultaneously by reversed-phase HPLC with multiwavelength detection. Clin. Chem..

[36] Korn J.M., Kuruvilla F.G., McCarroll S.A., Wysoker A., Nemesh J., Cawley S., Hubbell E., Veitch J., Collins P.J., Darvishi K. (2008). Integrated genotype calling and association analysis of SNPs, common copy number polymorphisms and rare CNVs. Nat. Genet..

[37] Mach 1.0- University of Michigan School of Public Health. http://www.sph.umich.edu/csg/abecasis/MACH/index.html.

[38] MMAP Documentation-University of Maryland School of Medicine. http://edn.som.umaryland.edu/mmap/index.php.

[39] McArdle P.F., O’Connell J.R., Pollin T.I., Baumgarten M., Shuldiner A.R., Peyser P.A., Mitchell B.D. (2007). Accounting for relatedness in family based genetic association studies. Hum. Hered..

[40] Grallert H., Dupuis J., Bis J.C., Dehghan A., Barbalic M., Baumert J., Lu C., Smith N.L., Uitterlinden A.G., Roberts R. (2012). Eight genetic loci associated with variation in lipoprotein-associated phospholipase a2 mass and activity and coronary heart disease: meta-analysis of genome-wide association studies from five community-based studies. Eur. Heart J..

[41] Racherla S., Arora R. (2012). Utility of Lp-PLA2 in lipid-lowering therapy. Am. J. Ther..

[42] Ziouzenkova O., Winklhofer-Roob B.M., Puhl H., Roob J.M., Esterbauer H. (1996). Lack of correlation between the α-tocopherol content of plasma and LDL, but high correlations for γ-tocopherol and carotenoids. J. Lipid Res..

[43] Gaughan L., Stockley J., Wang N., McCracken S.R., Treumann A., Armstrong K., Shaheen F., Watt K., McEwan I.J., Wang C. (2011). Regulation of the androgen receptor by SET9-mediated methylation. Nucleic Acids Res..

[44] Schulz W.A., Hatina J. (2006). Epigenetics of prostate cancer: beyond DNA methylation. J. Cell. Mol. Med..

[45] Varambally S., Dhanasekaran S.M., Zhou M., Barrette T.R., Kumar-Sinha C., Sanda M.G., Ghosh D., Pienta K.J., Sewalt R.G., Otte A.P. (2002). The polycomb group protein EZH2 is involved in progression of prostate cancer. Nature.

[46] Vieira F.Q., Costa-Pinheiro P., Ramalho-Carvalho J., Pereira A., Menezes F.D., Antunes L., Carneiro I., Oliveira J., Henrique R., Jeronimo C. (2013). Deregulated expression of selected histone methylases and demethylases in prostate carcinoma. Endocr. Relat. Cancer.

[47] Holzapfel N.P., Holzapfel B.M., Champ S., Feldthusen J., Clements J., Hutmacher D.W. (2013). The potential role of lycopene for the prevention and therapy of prostate cancer: From molecular mechanisms to clinical evidence. Int. J. Mol. Sci..

[48] Ross A.B., Vuong le T., Ruckle J., Synal H.A., Schulze-Konig T., Wertz K., Rumbeli R., Liberman R.G., Skipper P.L., Tannenbaum S.R. (2011). Lycopene bioavailability and metabolism in humans: An accelerator mass spectrometry study. Am. J. Clin. Nutr..

[49] Moran N.E., Cichon M.J., Riedl K.M., Grainger E.M., Schwartz S.J., Novotny J.A., Erdman J.W., Clinton S.K. (2015). Compartmental and noncompartmental modeling of 13C-lycopene absorption, isomerization, and distribution kinetics in healthy adults. Am. J. Clin. Nutr..

