# Supplementary material for: A Common Variant in the SETD7 Gene Predicts Serum Lycopene Concentrations
Source: Nutrients. 2016 Feb 6;8(2):82. doi: 10.3390/nu8020082 (PMC4772045; doi:10.3390/nu8020082)
Supplement: Supplementary file 1 [file nutrients-08-00082-s001.docx]

Supplementary Materials: A Common Variant in the SETD7 Gene Predicts Serum Lycopene Concentrations

Christopher R. D’Adamo, Antonietta D’Urso, Kathleen A. Ryan, Laura M. Yerges-Armstrong, Richard D. Semba, Nanette I. Steinle, Braxton D. Mitchell, Alan R. Shuldiner and Patrick F. McArdle

6-Day Controlled Diet: Full Menu

MONDAY AND THURSDAY MENU

**Breakfast:**

Kellogg’s Corn Flakes (1 box) 25 grams

Whole milk (1 cup)

Canned peaches in light syrup (1/2 cup) 131 grams

Whole-wheat bread (2 slices) 56 grams

Butter (2 pats) 14 grams

Hard-boiled egg (1) 50 grams

Pork sausage patty (1) 57 grams

Orange juice (1/2 cup)

Jelly (2 packets) 28 grams

Tea or coffee (1 teabag or 1 coffee sachet)

**Lunch:**

Broth: Herb-Ox Chicken Bouillon packet (1) 6 grams

Hot water (1 cup)

Whole-wheat bread (2 slices) 56 grams

Mayonnaise (2 packets) 24 grams

Turkey breast cold cut (80 grams)

American cheese (0.75 oz slice) 21 grams

Salad: Lettuce (1 cup) 55 grams

Tomato (1 small) 91 grams

Ranch salad dressing (1 packet) 12 grams

Fresh apple 138 grams

Orange juice (1/2 cup or 4 fluid oz)

Salt packet (1) or 1/2 salt tablet

**Dinner:**

Herbed pork loin or chicken breast (130 grams)

Mr. Spice sauce (1–2 tablespoons mix with 1 Herb-Ox chicken bouillon sachet)

Sweetened applesauce (1/2 cup) 127 grams

Rotini cooked with salt (3/4 cup) 100 grams

Meatless tomato sauce (1/2 cup) 120 grams

Peas (3/4 cup) 108 grams

Carrot (1/2 cup) 55 grams

Butter (2 pats) 14 grams

Whole-wheat bread (2 slices) 56 grams

Chocolate pudding, ready-to-eat (7.5 oz container) 131 grams

Orange juice (1/2 cup)

Salt packet (1) or 1/2 salt tablet (0.5 grams)

**Snack:**

Potato chips (salted, 1 oz bag) 28 grams

Sugar cookies (2) 32 grams

TUESDAY AND FRIDAY MENU

**Breakfast:**

Raisin bran (1 box) 52 grams

1% milk (1 cup)

Fresh fruit 127 grams

Whole-wheat bread (2 slices) 56 grams

Butter (2 pats) 14 grams

Hard-boiled egg (1) 50 grams

Pork sausage patty (1) 57 grams

Orange juice (1/2 cup)

Jelly (1 packet) 14 grams

Tea or coffee (1 teabag or 1 coffee sachet)

**Lunch:**

Navy bean soup (1 cup)

Whole-wheat bread (2 slices) 56 grams

Mayonnaise (1 packet) 12 grams

Turkey breast cold cut (regular, 80 grams)

American cheese (1 slice 3/4 oz) 21 grams

Salad: Lettuce (1 cup) 55 grams

Tomato (1 small) 91 grams

Ranch/Thousand Island dressing (2 packets) 24 grams

Pears in light syrup (1/2 cup) 133 grams

Potato chips (salted, 1 oz bag) 28 grams

Orange juice (1/2 cup or 4 fluid oz) 124 grams

Salt packet (1) or 1/2 salt tablet

**Dinner:**

Chicken Pie (200 grams and mix with 1 Herb-Ox chicken bouillon sachet)

Scalloped potato (170 grams

Corn (1/2 cup) 82 grams

Peas (1/2 cup) 80 grams

Whole-wheat bread (1 slice) 28 grams

Apple crisp (150 grams)

Orange juice (1/2 cup)

Salt packet (1) or 1/2 salt tablet (0.5 grams)

**Snack:**

Sweetened applesauce (1/2 cup) or a fresh apple

Peanut butter sandwich crackers (Packet of 3 sandwich crackers) 50 grams

WEDNESDAY AND SATURDAY MENU

**Breakfast:**

Kellogg’s Frosted Shredded Wheat (1 box) 52 grams

Whole milk (1 cup)

Whole-wheat bread (2 slices) 56 grams

Butter (2 pats) 14 grams

Bacon (3 slices) 25 grams

Hard-boiled egg (1) 50 grams

Orange juice (1/2 cup)

Salt packet (1) or 1/2 salt tablet

Jelly (2 packets) 28 grams

Tea or coffee (1 teabag or 1 coffee sachet)

**Lunch:**

Broth: Herb-Ox chicken bouillon packet (1) 6 grams

Hot water (1 cup)

Sandwich:

Whole-wheat bread (2 slices) 56 grams

Mayonnaise (2 packets) 24 grams

American cheese (2 slices) 42 grams

Ham (2 slices) 80 grams

Salad: Lettuce (1 cup) 73 grams

Tomato (1 small) 91 grams

Ranch/Thousand Island salad dressing (2 packets) 24 grams

Peaches in light syrup (1 cup) 262 grams

Orange juice (1/2 cup)

**Dinner:**

Meatloaf (130 grams) served with 2 tbsp of BBQ sauce (34 grams)

Mashed potatoes mixed with 1 Herb-Ox chicken bouillon sachet (11 grams)

Green beans (1/2 cup) 67 grams

Carrot (1/2 cup) 55 grams

Sweetened applesauce (1 cup) 254 grams

Butter pats (2) 14 grams

Angel food cake (60 grams)

Orange juice (1/2 cup)

Salt packet (1) or 1/2 salt tablet (0.5 grams)

**Snack:**

Potato chips (salted, 1 oz) 28 grams

Sugar cookies (2) 32 grams
